# THE PREVALENCE OF ORTHOSTATIC HYPOTENSION AMONG HOSPITALIZED OLDER ADULTS AT RISK FOR FALLING

**DOI:** 10.1093/geroni/igz038.3120

**Published:** 2019-11-08

**Authors:** Denise L Lyons, Kathleen Schell

**Affiliations:** 1 Christiana Care Health system, Newark, Delaware, United States; 2 University of Delaware, Newark, Delaware, United States

## Abstract

Orthostatic hypotension (OH) may cause falls in hospitalized older adults. OH is a sustained drop of at least 20 mm Hg for systolic blood pressure or at least 10 mm Hg for diastolic blood pressure when changing position from supine to sitting, sitting to standing, or supine to standing. A recent systematic review revealed an inconsistent relationship between OH and falls. Orthostatic vital signs (OVS) measurement is often included in fall prevention initiatives. Some experts suggest that the time required to collect OVS and the possibility of measurement inaccuracy by nurses make this bedside screening unnecessary. The study aims were to determine: 1) the prevalence of OH, 2) if those older adults with documented OH experienced falls, and 3) the influence of medications known to be associated with OH and falls. Medication categories included antihypertensives, dopamine agonists, antipsychotics and antidepressants. A retrospective chart review was conducted on a convenience sample of 8,474 older adults on two Acute Care of the Elderly units at a large health system in the mid-Atlantic between 2015 and 2018. Results were determined using contingency tables and Chi-square analysis. More complex relationships were pursued using log linear models. The overall OH prevalence was 46.9% at some point during their hospital stay. Over the three years, 68 patients of whom 62% were hypotensive (p=.034). There was no statistical association of OH with medications or co-morbidities. The results demonstrate that although prevalent in almost half of this sample, orthostatic hypotension did not lead to falls.

